# Multiple evanescent white dot syndrome (MEWDS): update on practical appraisal, diagnosis and clinicopathology; a review and an alternative comprehensive perspective

**DOI:** 10.1186/s12348-021-00279-7

**Published:** 2021-12-18

**Authors:** Ioannis Papasavvas, Alessandro Mantovani, Ilknur Tugal-Tutkun, Carl P. Herbort

**Affiliations:** 1 Retinal and Inflammatory Eye Diseases, Centre for Ophthalmic Specialized Care (COS), Clinic Montchoisi Teaching Centre, Rue Charles-Monnard 6, CH-1003 Lausanne, Switzerland; 2 Department of Ophthalmology, Valduce Hospital, Como, Italy; 3 Department of Ophthalmology, Istanbul University, Istanbul Faculty of Medicine, Istanbul, Turkey

**Keywords:** MEWDS, ICGA, PICCPs, BL-FAF

## Abstract

**Background:**

Multiple evanescent white dot syndrome (MEWDS) is a rare inflammatory eye condition affecting the outer retina as a consequence of choriocapillaris non perfusion. The pathophysiology of MEWDS will be discussed based clinical appraisal and on multimodal imaging appraisal.

**Methods:**

Narrative review and perspective opinion.

**Results:**

Literature review results helped us to put forward (1) the specific symptomatology (decreased/blurred vision, photopsia, subjective scotomas), (2) the ill-asserted character of clinical findings (foveal granularity, white dots in fundoscopy), (3) and the crucial importance of multimodal imaging with the diagnostic triad of ICGA hypofluorescent areas, BL-FAF hyperautofluorescent areas and loss/damage of IS/OS-ellipsoid zone on SD-OCT that characterise the disease and can practically help the clinician to diagnose MEWDS. A comprehensive alternative perspective of the disease was formulated.

**Conclusions:**

The bulk of evidence that we are presenting in this review, thanks to new performing non-invasive and invasive imaging modalities, is sufficiently compelling to consider MEWDS as a primary choriocapillaritis/inflammatory choriocapillaropathy. Multimodal imaging allows the clinician to diagnose MEWDS with a high level of certainty and ensures a precise follow-up.

## Introduction

Multiple evanescent white dot syndrome (MEWDS) is a rare posterior uveitis, characterised by numerous pale whitish dots seen in the posterior pole and the midperiphery [1]. As its name indicates the appearance of the dots is limited in time and may not be present when the patient consults. In the post-acute phase, a granular aspect of the fovea persists [2]**.** Patients complain of subjective scotomas and photopsia. In some cases, up to 50%, the ocular disease is preceded by a flu-like viral episode [1]. Except rare reported bilateral forms, it is a unilateral disease and affects young to middle-aged adults, being predominant in women and in myopic patients [3]. It is best diagnosed by indocyanine green angiography (ICGA) identifying scattered areas of hypofluorescence [4–6] or by blue-light fundus autofluorescence (BL-FAF) showing scattered hyper-autofluorescent areas co-localising with the ICGA hypofluorescent areas [7]. Spectral domain optical coherence tomography (SD-OCT) shows damage to the outer segments of the photoreceptor line [8]**.** Visual loss can be minor to very pronounced, depending on the areas involved and the severity of the process [9]. Visual field testing can show faint to pronounced scotomas with an often enlarged blind spot [10, 11]. Visual function is restored without treatment within 8–10 weeks and the disease does usually not recur. If there is a recurrence, idiopathic multifocal choroiditis has to be suspected, as these two diseases can overlap [12]. MEWDS is considered to be on the benign end of the primary choriocapillaritis entities, as it is usually reversible without treatment [13].

## Historical aspects and nomenclature

Between the late 1960s and the early 1990s, many chorioretinal diseases were described and characterised, starting with acute posterior multifocal placoid pigment epitheliopathy (APMPPE) reported by Gass in 196 8[14]. Multifocal choroiditis, now called idiopathic multifocal choroiditis (MFC) was reported in 1969 by Krill [15] and in 1973 by Dorsh and Nozik [16]. In 1980 birdshot retinochoroiditis was described [17], in 1990 acute syphilitic posterior placoid chorioretinitis (ASPPC) [18] and in 1992 acute zonal occult outer retinopathy (AZOOR) [19] to cite only the more important entities and leaving out the many more sub-entities. The descriptions of these conditions were very accurate, based on precise phenomenological observation of signs and precise follow-up. However, in the absence of multimodal imaging, the pathophysiological explanations were often conjectural and left the clinicians doubtful and uncomfortable about disease mechanisms. An example of such an erroneous interpretation is APMPPE, a disease attributed to the retinal pigment epithelium (RPE) by Gass and correctly interpreted as a choriocapillaritis by Deutman in 1972 who called the disease acute multifocal ischaemic choriocapillaritis (AMIC) [20]. Therefore, when in 1995 an article proposed to assemble these diseases within the group of the “white dot syndromes” (WDS), this terminology was quickly adopted by the ophthalmological community at large [21]. Today we know that disease mechanisms in this group differ substantially and there is no reason to use this potpourri classification any longer.

MEWDS was part of these newly described entities during the glorious late 1960s to the early 1990s. In 1984, Lee Jampol, Paul Sieving and colleagues published two remarkable articles precisely describing the clinical and electrophysiological characteristics of the disease [22, 23]. The authors did not venture to give a pathophysiological hypothesis but gave a very comprehensive and detailed clinical description. Unfortunately, MEWDS was also included in the WDS group. With the availability of more performing imaging modalities such as ICGA and SD-OCT it was possible to demonstrate that the disease was caused by vaso-occlusive problems at the level of the inner choroid producing ischaemia in the outer retina and damage to the outer segments of the photoreceptors. By its disease mechanism, namely inflammatory choriocapillaris non-perfusion, MEWDS distinguishes itself from other diseases classified in the WDS group, such as birdshot chorioretinitis, characterised by choroidal stromal infiltration. The WDS terminology is therefore inappropriate, and these diseases should, more appropriately, be classified according to their disease mechanisms including choriocapillaritis, stromal choroiditis and other mechanisms [24].

In 1988, a group from San Francisco described an entity which they called *“Acute idiopathic blind spot enlargement. A big blind spot syndrome without optic disc edema”* (AIBSE) [25]**,** later identified as an expression of MEWDS by the group of Donald Gass [26]. AIBSE could be linked to MEWDS by performing ICGAs in AIBSE patients without fundus white dots that showed hypofluorescent areas of choriocapillaris non-perfusion typical of MEWDS [27]. In addition to AIBSE cases, many typical MEWDS cases showed an enlarged blind spot [28, 29].

More recently, despite the ICGA signs typical of MEWDS hypofluorescence indicating choriocapillaris non-perfusion, it was hypothesised that MEWDS was a primary photoreceptor disease, a conjecture hard to take as explained hereunder [30].

Classification criteria were presented recently by the SUN group. Unfortunately, similar to classification criteria of other posterior uveitis entities by the group, they are of limited use in practice, as they neither included ICGA nor BL-FAF in the proposed criteria [31].

## Clinical findings and disease course

The typical patient with MEWDS is a myopic woman between the ages of 20 and 40 who presents with acutely diminished visual acuity, photopsias, and temporal visual field defects in one eye following an episode of flu-like symptoms. It is a rare disease accounting for 1.24% of uveitis diagnoses in our setting. Ocular examination typically reveals trace vitreous cells, numerous isolated or confluent yellow-white spots and dots ranging in size from 100 μm to more than 200 μm, at the level of the RPE or deep retina, mostly concentrated in the paramacular and peripapillary area and randomly scattered in the mid-peripheral retina, foveal granularity, and mild optic disc inflammation in the involved eye. The natural evolution of typical MEWDS is characterized by spontaneous resolution of fundus findings and recovery of visual function within several weeks. The clinical diagnosis of MEWDS is confirmed by multimodal imaging at presentation and during follow-up.

Table 1 shows demographic and clinical data from the original description of 11 MEWDS cases by Jampol et al. [22] and data from 3 large series published in the last decade [3, 32, 33]. In contrast to the original description of unilateral monophasic nature of MEWDS, recent large cohorts have shown simultaneous or sequential involvement of both eyes in up to 10% of patients and recurrences in up to 14% [3, 32, 33]. Mild involvement of the other eye could be detected by ICGA or OCT imaging [3]. Foveal granularity was documented in 70–94% of cases, consistent with the original description; on the other hand, a diagnosis of MEWDS could still be made in the absence of characteristic white dots in recent cohorts [3, 33]. In fact, foveal granularity can be the presenting finding in patients who have suggestive symptoms of MEWDS but do not have white dots on fundus examination [2, 34]. The clinician can see patients with a normal fundus despite the very recent onset of symptoms. Because of the transient nature of white dots, a characteristic finding is a yellowish macula with granularity which can last longer and could be the only finding seen by the ophthalmologist at the time of examination in the post-acute phase. An incomplete visual recovery is another atypical feature in MEWDS patients.

**Table 1:** Demographic and clinical data from MEWDS case series

Author/ year	No	Demographic and clinical features at presentation								Course	
		Mean Age (y)	F gend %	Preceding flulike illness %	Myopia %	UI %	WD %	Foveal granularity %	OD infl %	MT to recovery (w)	Recurrence %
Jampol 1984 [22]	11	28	90.9	45	N/A	100	100	90.9	N/A	7	0
Marsiglia 2016 [3]	34	28.7	76.4	8.8	85.7	94.1	85.3	94.1	79.4	10	8.8
Bosello 2020 [32]	51	29.6	80.4	23.5	N/A	90.2	N/A	70.3	16.2	N/A	11.7
Ramakri. 2021 [33]	73	35.2	79.5	23	48	99	92	74	52	N/A	14

*No* number of patients, *UI* unilateral involvement, *MT* mean time.

*Y* years, *WD* white dots, *w* weeks.

*F gend* female gender, *OD infl* Optic disc inflammation, *N/A* non applicable

Although MEWDS could be considered a “common cold” of the retina because of the transient nature of morphologic and functional changes, [35] Bosello et al. [32] have reported recovery of visual acuity to 0.0 LogMAR or better in 80% of MEWDS patients; and they found that poor initial visual acuity and young age were associated with incomplete visual recovery. Even though foveal granularity persisted at 3 months in 40% of their patients, it did not seem to be associated with poor recovery of visual function [32]. However, hyperfluorescent disc on fluorescein angiography (FA) at presentation was more frequent in patients with incomplete visual recovery (75% versus 42%) [32]. Patients who developed choroidal neovessels (CNVs) as a cause of poor visual outcome had been already excluded from their analysis [32]. Marsiglia et al. [3] have reported persistent peripapillary atrophy (23.5%) and multifocal pigmentary changes (5.8%) as sequelae of MEWDS in 34 patients who had presented with a typical episode and showed a mean recovery of visual acuity from 0.41 LogMAR to 0.03 LogMAR. Hamed et al. [10] were the first to show that the enlargement of the blind spot may persist for several months after the resolution of fundus lesions and recovery of visual acuity in some MEWDS patients.

Focal choroidal excavation and macular or peripapillary CNVs are rare complications of MEWDS and may appear several months or years after the resolution of white dots [36–40]. There are also reports of patients who present concurrently with CNVs and MEWDS or develop characteristic MEWDS lesions later during follow-up [41–44]. In such cases, CNVs may be an inaugural sign of MEWDS or a trigger of the MEWDS phenotype [42–44].

## Visual field testing and imaging investigation of MEWDS

### Visual field testing

**Fig. 1:**
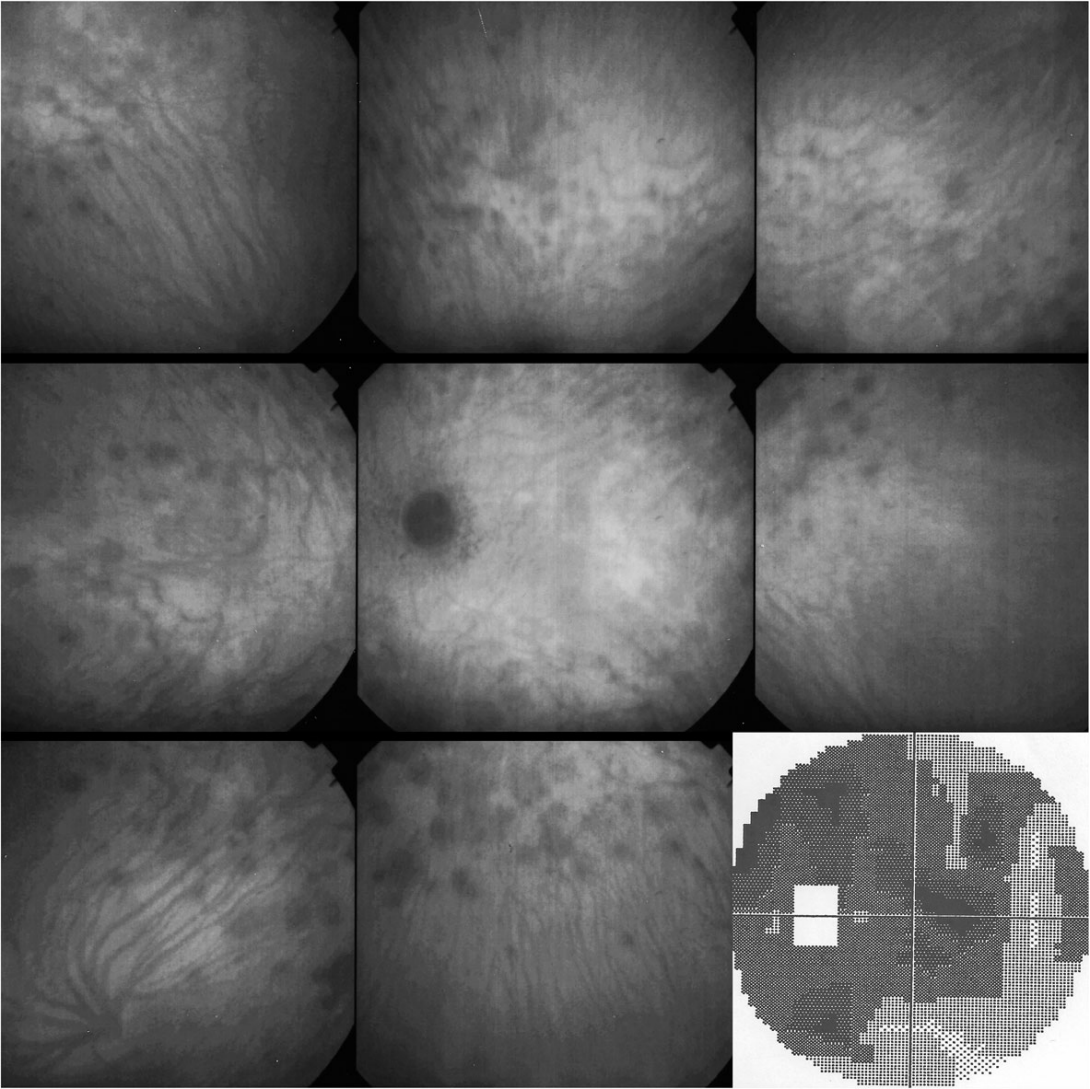
MEWDS patient referred for suspected retrobulbar neuritis with severe visual field (VF) impairment. ICGA shows numerous hypofluorescent areas and a peripapillary hypofluorescent annulus explaining the severe VF deficit. VF returned to normal after 8 weeks

Reports on isolated MEWDS cases usually signal visual field impairment but reports of larger series with specific analysis of visual field changes are scarce [45]. Visual field testing and microperimetry show that functional consequences can be quite diverse, going from large central scotomas to absence of visual field impairment. In our series of 20 patients, mean MD (mean defect) amounted to 6.1 ± 2.8 (normal < 2) with values from 0.3 to 21.2. Microperimetry was more appropriate in determining reduction of function. In our experience, 8 out of 9 tested patients had a significantly decreased microperimetry score in the MEWDS eye compared to the contralateral normal eye (357 ± 106.7 versus 465.3 ± 47.8) (*p* < 0.016, Student’s t-test) (Fig. 1). Obviously the extent of visual field damage depends on the severity of the MEWDS episode that can reach from subtle to very pronounced, which is the case for all choriocapillaritis entities that can have different grades of involvement depending on the importance of the choriocapillaris/choroidal vaso-occlusive process.

#### Multimodal imaging

Multimodal imaging has become the mainstay of diagnosis of MEWDS. In our diagnostic arsenal we have non-invasive methods (BL-FAF, SD-OCT, VF, OCT-A) and invasive methods (FA, ICGA) to detect pathological lesions in the retina and choroid.

FA, classically used in posterior uveitis has minimal value-added potential in MEWDS. The first substantial progress in imaging MEWDS was achieved by ICGA in the mid-1990s. This was followed by BL-FAF clearly identifying the same MEWDS lesions as those seen by ICGA, but in a non-invasive fashion, and SD-OCT morphologically demonstrating loss or damage of the photoreceptor outer segments.

### Laser flare photometry/intraocular inflammation

MEWDS is not associated with detectable anterior inflammation, even when laser flare photometry is used the sparse subclinical inflammation is low. In our setting, the mean level of flare in a series of 20 MEWDS patients amounted to 6.1 ± 2.8 (normal values 4–6 ph/ms) with 12.4 ph/ms being the highest value. In 6 of 20 patients slight posterior vitritis was noted.

### Fundus photography

**Fig. 2:**
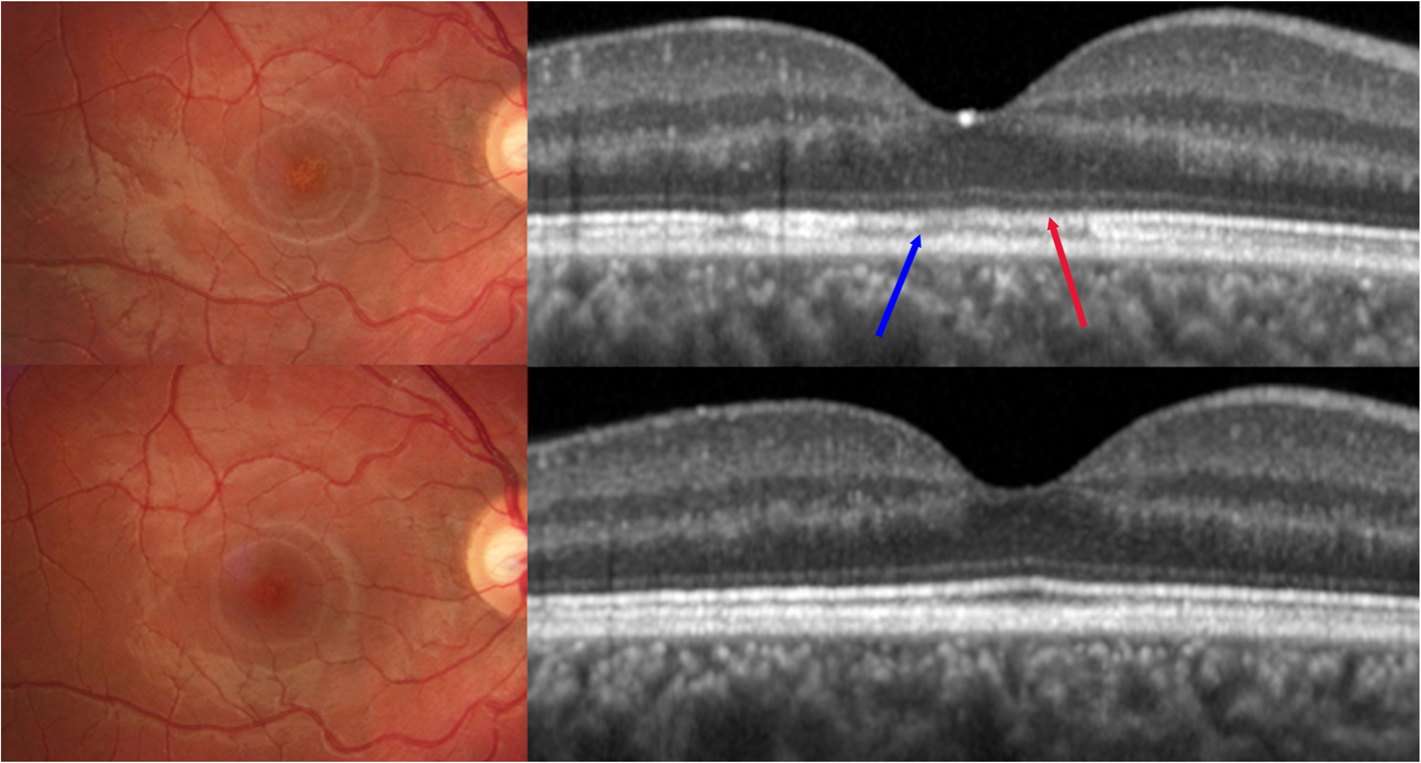
Fundus / ICGA / BL-FAF signs in a typical MEWDS case. Faint fundal white dots are visible at presentation (top left), quickly disappearing on day 2 (D2) (middle left) and barely visible on day 3 (D3) (bottom left). BL-FAF hyperautofluorescence (top right) and ICGA hypofluorescence (bottom right) clearly identify the diseased areas

**Fig. 3:**
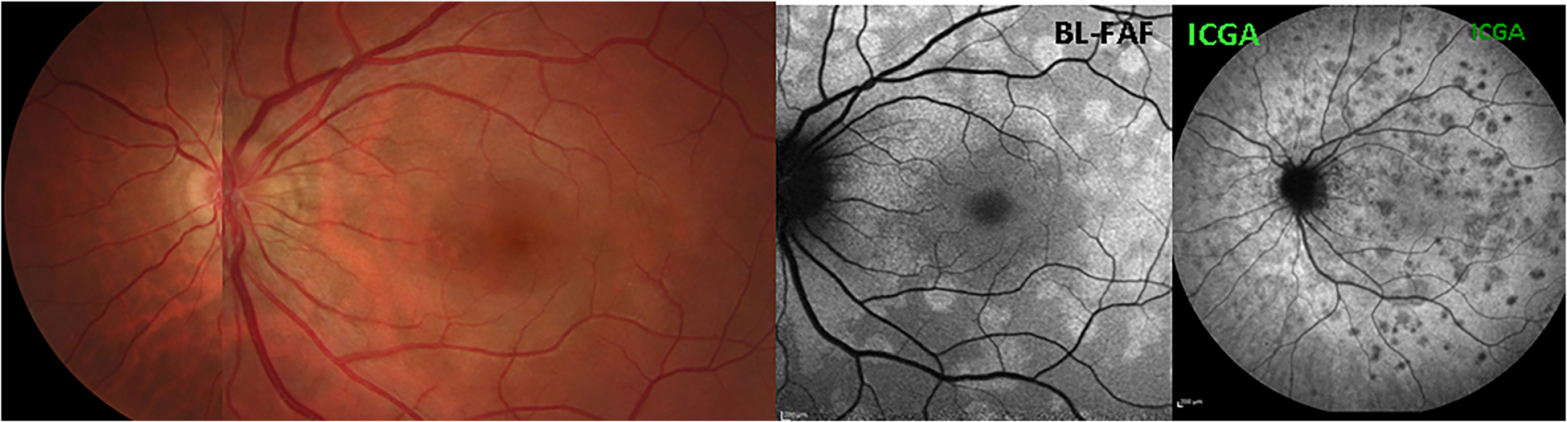
Fundus images in MEWDS. Fundus, BL-FAF and ICGA in a MEWDS patient that consulted 3 days after symptoms of photopsias and subjective scotomas. Fundus showed absence of white dots and foveal granularity. BL-FAF (middle image) showed numerous prominent areas of hyperautofluorescence, while ICGA late phase frame revealed numerous dark dots co-localising with BL-FAF hyperautofluorescent areas

**Fig. 4:**
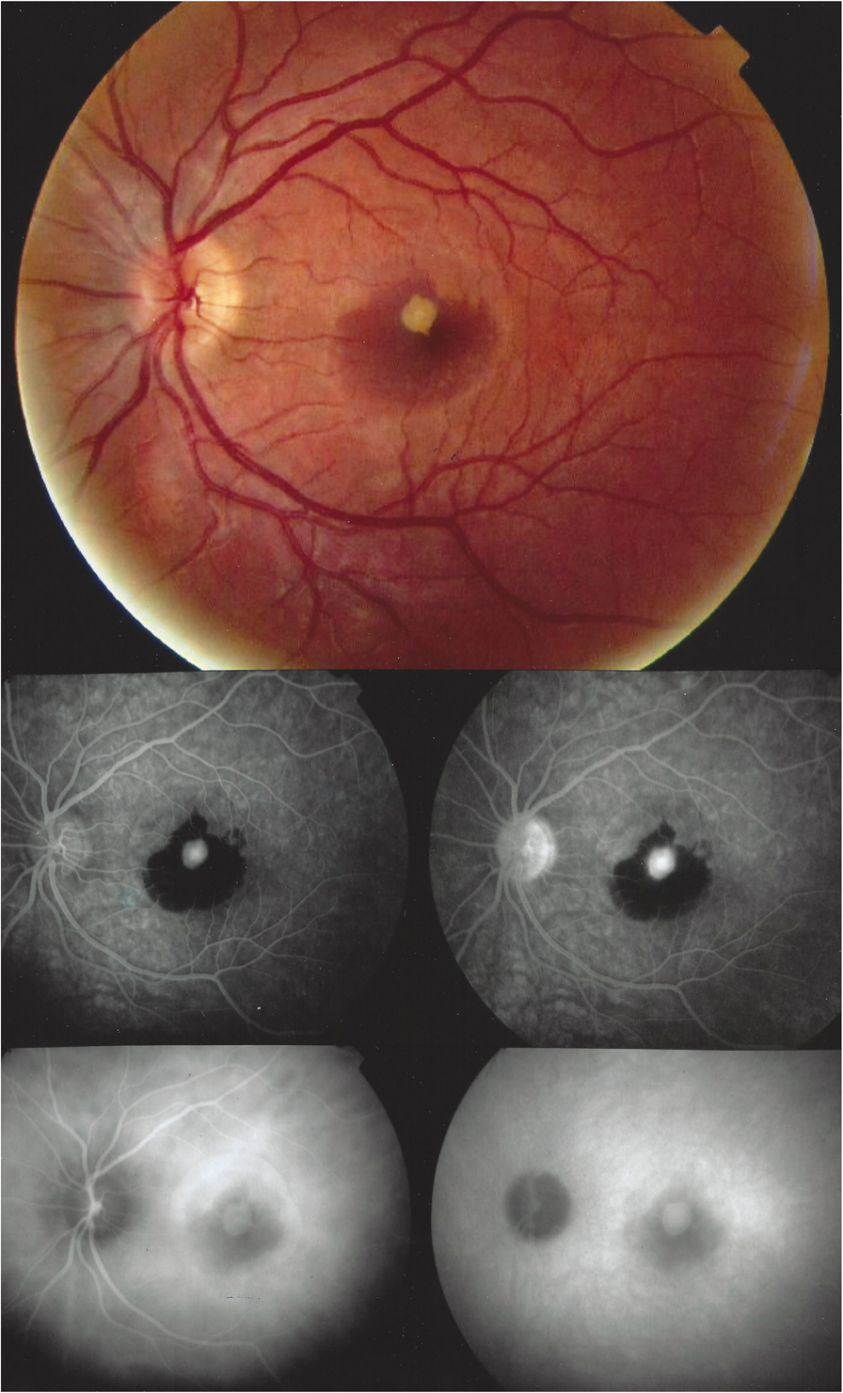
Inflammatory CNVs in a 16-year-old youngster. Fundus (top) shows the CNVs with an intraretinal haemorrhage. FA (middle) shows the bright hyperfluorescent CNVs, also hyperfluorescent on ICGA (bottom). A few days later, a repeat ICGA showed the typical ICGA signs of MEWDS (see Fig. 9)

MEWDS fundus examination usually displays multiple, small whitish dots located around the optic disc or scattered throughout the posterior pole and the mid-periphery. In addition to the multiple white lesions, another typical feature is the granular appearance of the fovea, which can be present even as a stand-alone feature (Fig. 2). Since the dots can disappear very rapidly, it is also possible that fundoscopy shows absence of pathology if the patient does not consult the clinician at an early stage [2] (Fig. 3). In our series white dots were recorded in only 6/20 (33.3%) patients indicating that it is a good disease defining criterion when present that however has a limited sensitivity, probably because patients present late. However, granularity of the fovea was noticed in 13/20 (65%) patients and represents a more reliable sign. Fundoscopic signs together with symptoms of photopsia and subjective scotomas are no more than diagnostic hints with diagnosis confirmed by the triad ICGA, BL-FAF and SD-OCT. Further fundoscopic signs can comprise images of CNVs and haemorrhages as shown on Fig. 4.

### Fluorescein angiography (FA)

**Fig. 5:**
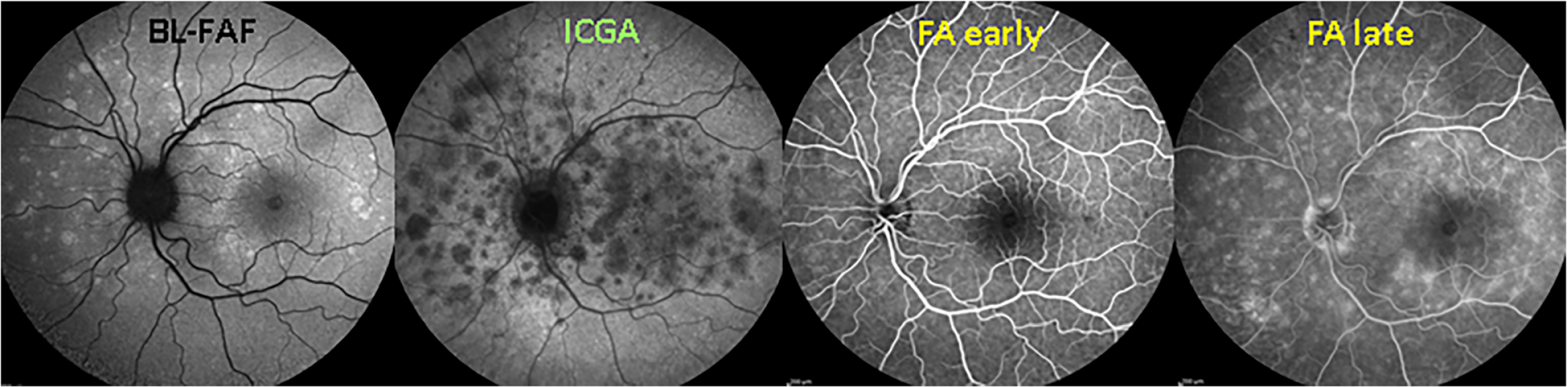
FA in MEWDS. FA signs in a MEWDS patient in the acute phase. BL-FAF picture (left) and ICGA (second from left) show typical disease signs. Early FA (second from right) shows very faint hyperfluorescence while late FA shows more distinguishable hyperfluorescent areas (right)

Fluorescein Angiography findings in MEWDS consist of hyperfluorescent patchy lesions that can be quasi absent or very minimal; these FA signs appear in the early phases but show themselves more clearly in the mid-to-late phases of the angiography. They can also be very pronounced in some cases (Fig. 5).

**Fig. 6:**
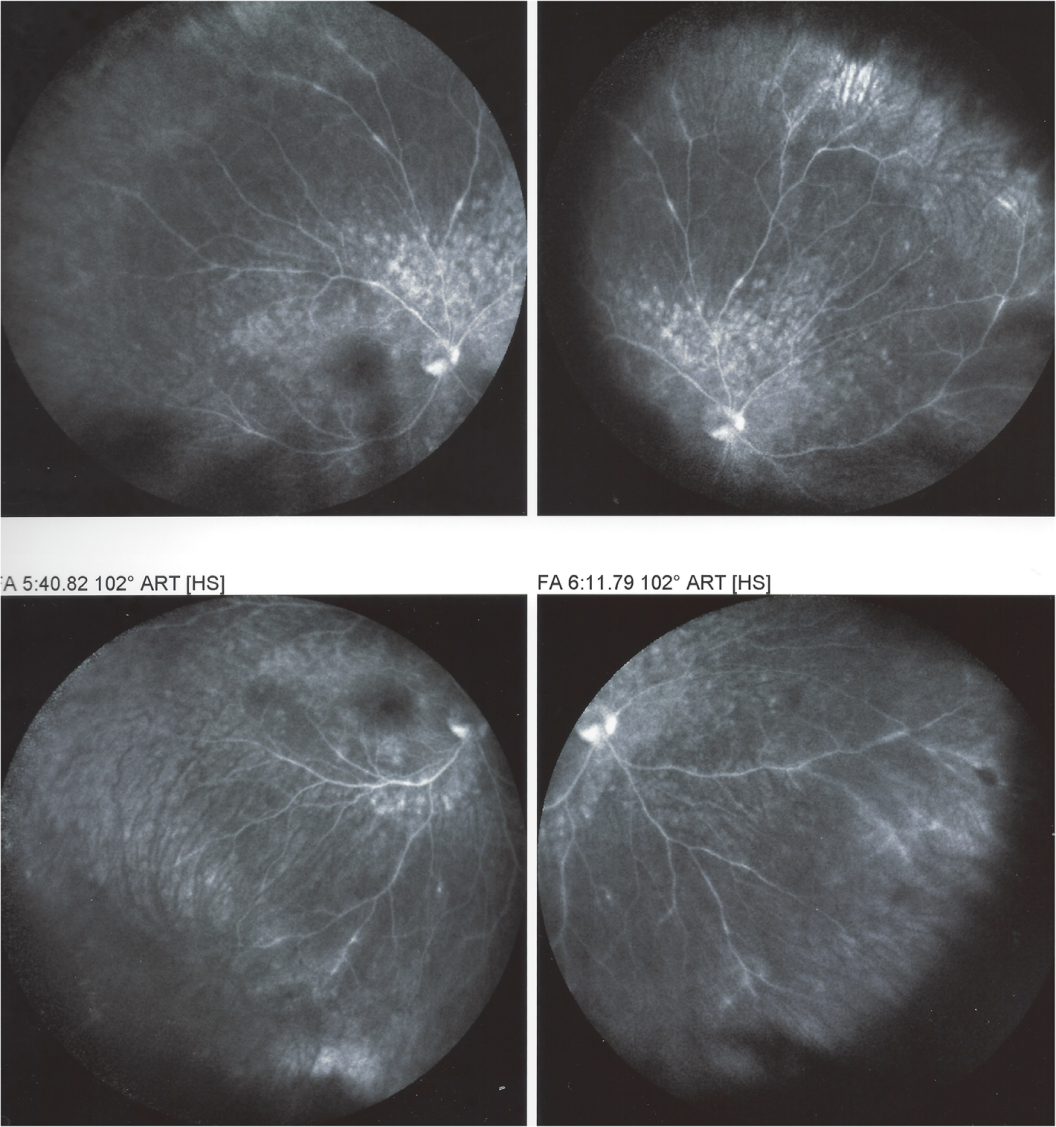
Retinal vasculitis in MEWDS. FA showing patchy hyperfluorescent area in the posterior pole as well as peripheral retinal vasculitis. (same patient as in Fig. 7)

The explanation for FA patchy hyperfluorescent areas is probably similar to what occurs in APMPPE or MFC where FA hyperfluorescence especially in the late phases can be pronounced in severe cases representing a reactive retinal vasodilatation and exudation secondary to outer retinal ischaemia caused by choriocapillaris hypo or non-perfusion [13, 46, 47]. Indeed, choroidal haemodynamic problems have been demonstrated in several studies [5, 6, 48–50]. Moreover, impaired choroidal perfusion was identified as a common denominator of choriocapillaritis entities including MEWDS [51]. As for APMPPE the degree of FA patchy hyperfluorescence depends on the severity of the vaso-occlusive process. Because of the discrete and sometimes absent FA signs, FA is of limited use in MEWDS. Additional signs found on FA are disc hyperfluorescence as well as peripheral retinal vasculitis sometimes described [52] (Fig. 6).

### Indocyanine green angiography (ICGA)

**Fig. 7:**
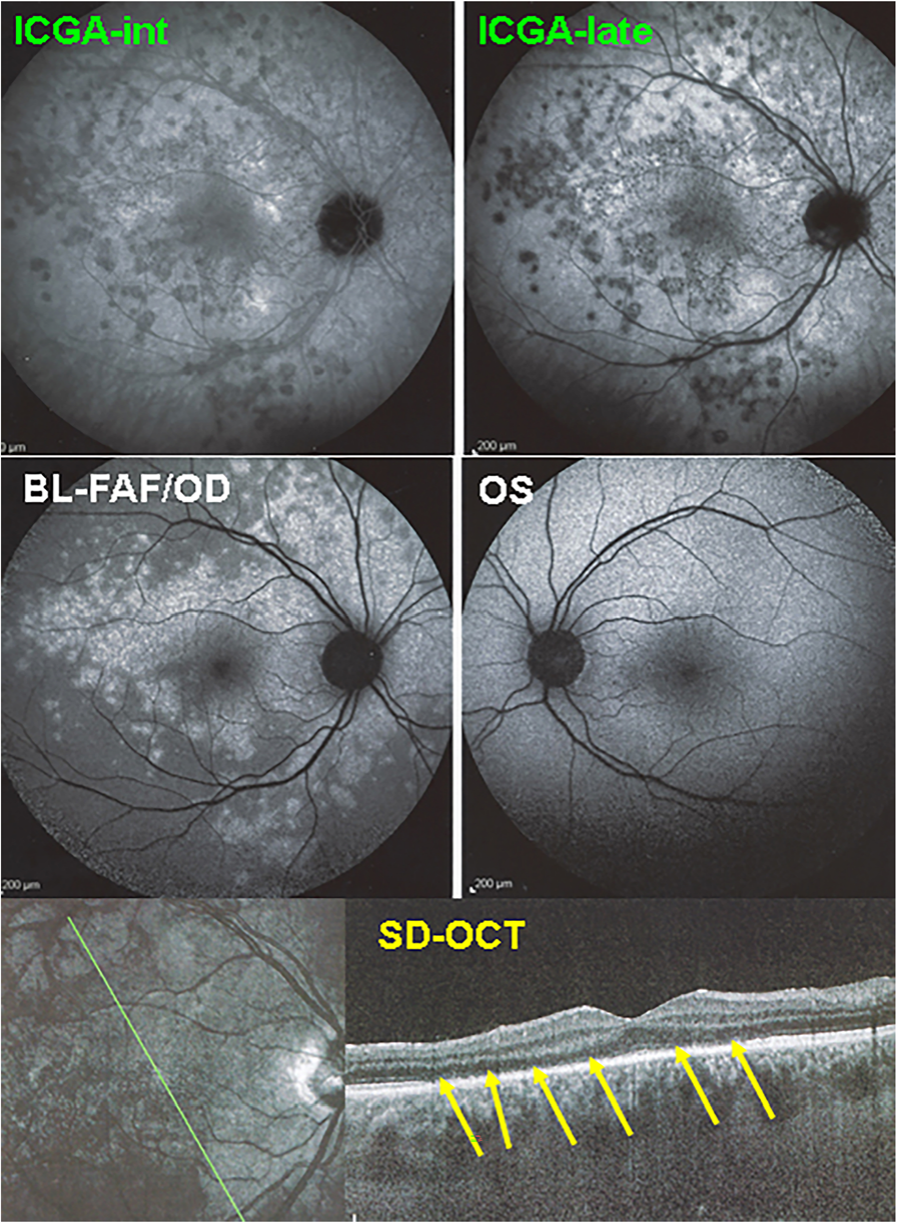
ICGA in MEWDS. ICGA hypofluorescence is present in the intermediate angiographic phase (top left) and more clearly detected in the late phase (top right). It co-localises with BL-FAF hyperautofluorescent areas (middle left) and corresponds to loss or damage of photoreceptor outer segments (bottom, yellow arrows)

Very quickly after ICGA started to be available and used at large the crucial importance of this imaging modality became apparent for MEWDS [4]. **ICGA** findings consist of patchy hypofluorescent areas in the posterior pole and in the mid periphery as well as around the optic disc. ICGA hypofluorescence is especially well visible in the late angiographic phase, which is speaking more for choriocapillaris hypoperfusion than for total non-perfusion (Fig. 7). This could also explain the usually benign course of the disease.

**Fig. 8:**
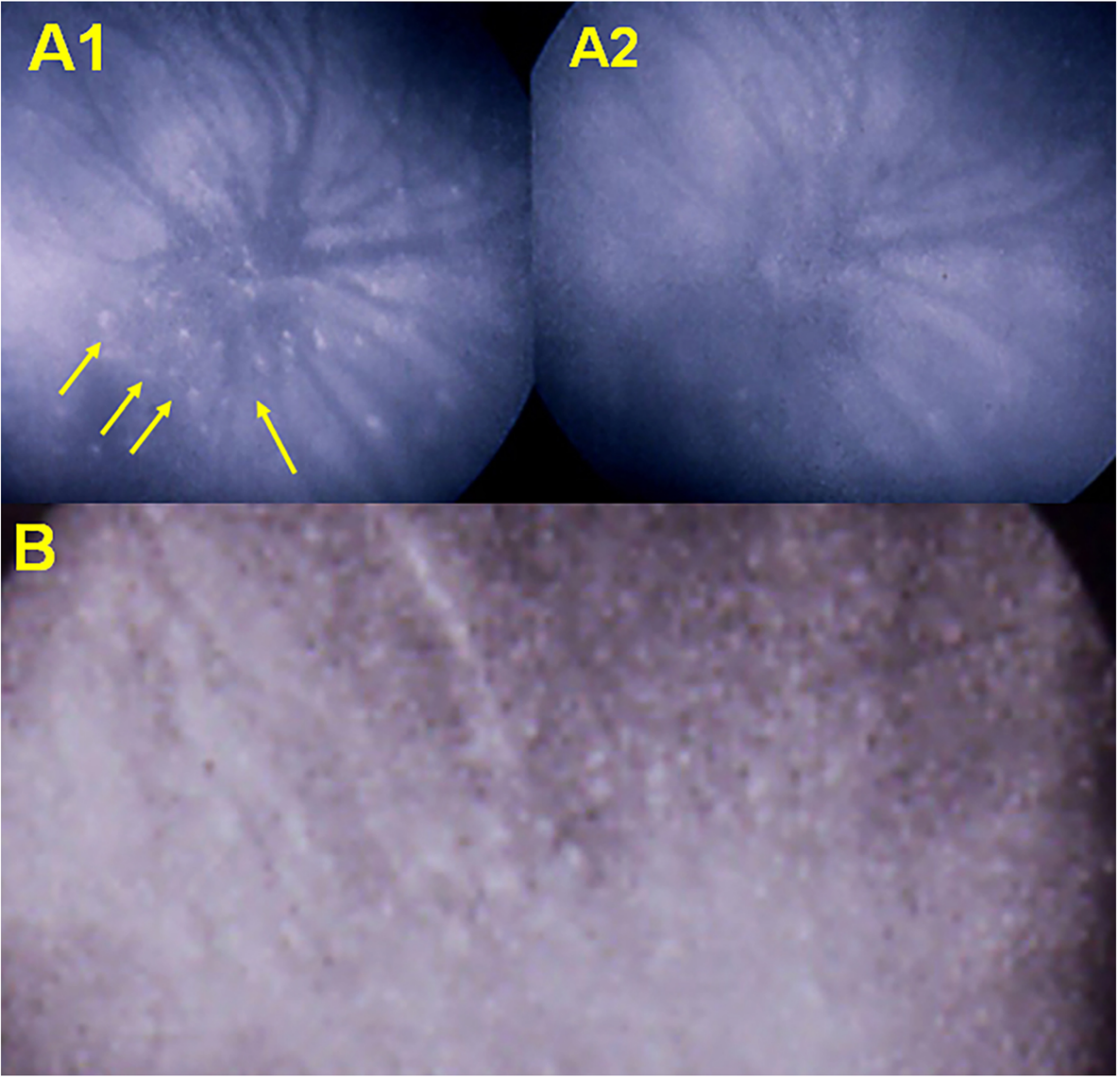
ICGA of peripheral pinpoints in granulomatous chorioretinitis. Diseased areas hyper-fix the ICG molecule and are not hypofluorescent as pretended by some. On the contrary they constitute hyperfluorescent pinpoint, in tuberculous chorioretinitis at presentation (A1) with disappearance after treatment (A2); and sarcoidosis chorioretinitis B where numerous diseased hyperfluorescent pinpoints are visible

The lesions exactly co-localise with BL-FAF hyper-autofluorescent areas and correspond to photoreceptor outer segment loss or damage on SD-OCT (Fig. 7). The only possible explanation for these hypofluorescent areas is hypo or non-perfusion of the choriocapillaris and cannot correspond to the alleged non-fixation of the ICGA molecule on hypothetically damaged RPE cells which is a pure conjecture. Indeed, an in-vitro study showed the opposite indicating increased infrared fluorescence in damaged RPE cells [53]. In clinical situations also, such as granulomatous chorioretinitis, diseased areas in the periphery accumulate ICGA and appear as hyperfluorescent pinpoints [54] (Fig. 8).

**Fig. 9:**
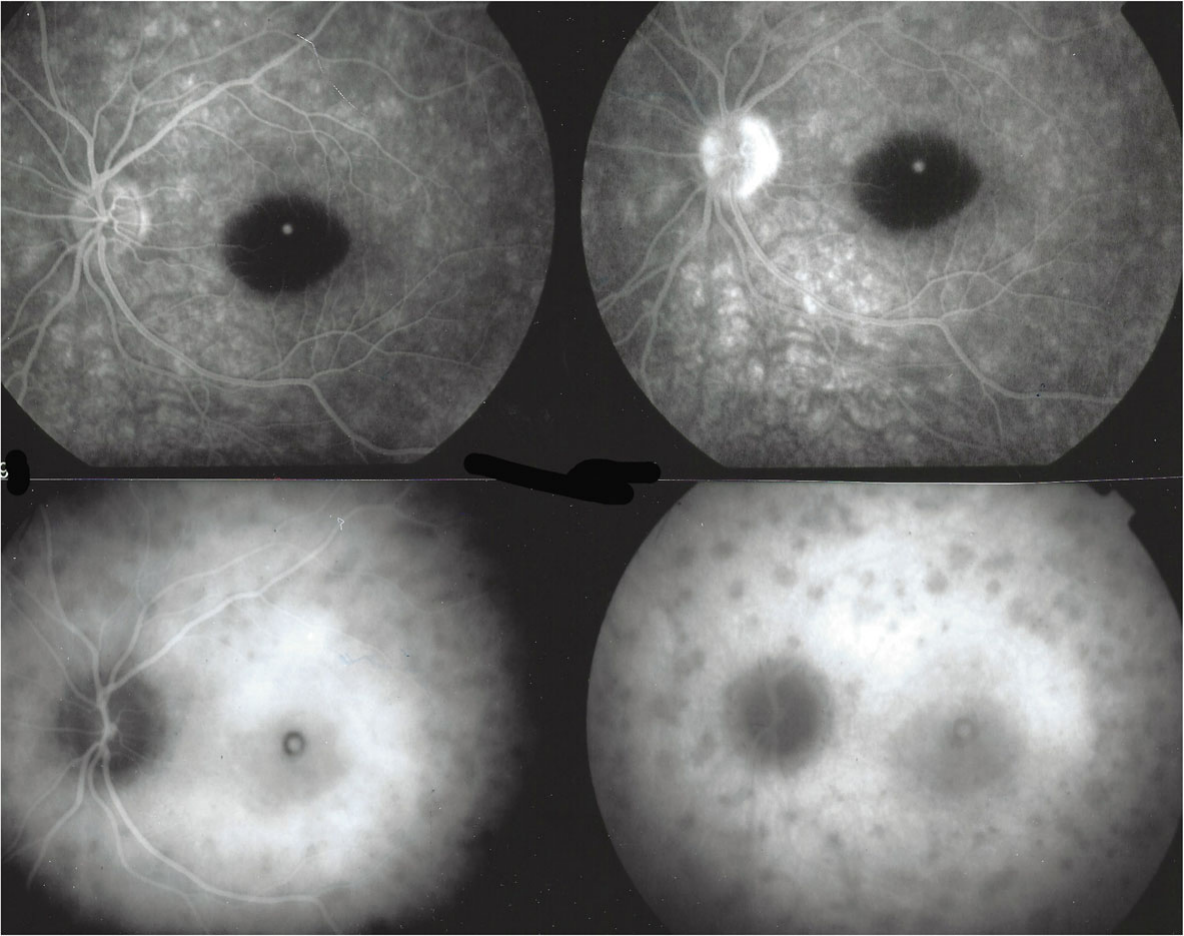
Inflammatory CNVs in a 16-year-old youngster before outbreak of MEWDS (same patient as Fig. 4). Ten days after intravitreal anti-VEGF injection, the CNVs are markedly reduced and appearance of slight FA hyperfluorescent areas (top) and typical areas of hypofluorescence on ICGA in the intermediate phase (bottom left) and more clearly visible in the late angiographic phase (bottom right)

ICGA, together with BL-FAF is by far the most important imaging modality to ascertain the diagnosis of MEWDS and is also the best way to establish the extent of the lesion process and to explain the severity of visual impact. It used to be the most useful follow-up parameter but is today advantageously replaced by BL-FAF, as it is a non-invasive method. ICGA is rarely useful to detect MEWDS as the origin of inflammatory CNVs (Figs. 4 & 9) [41]**.**

### Spectral-domain optical coherence tomography (SD-OCT)

**Fig. 10:**
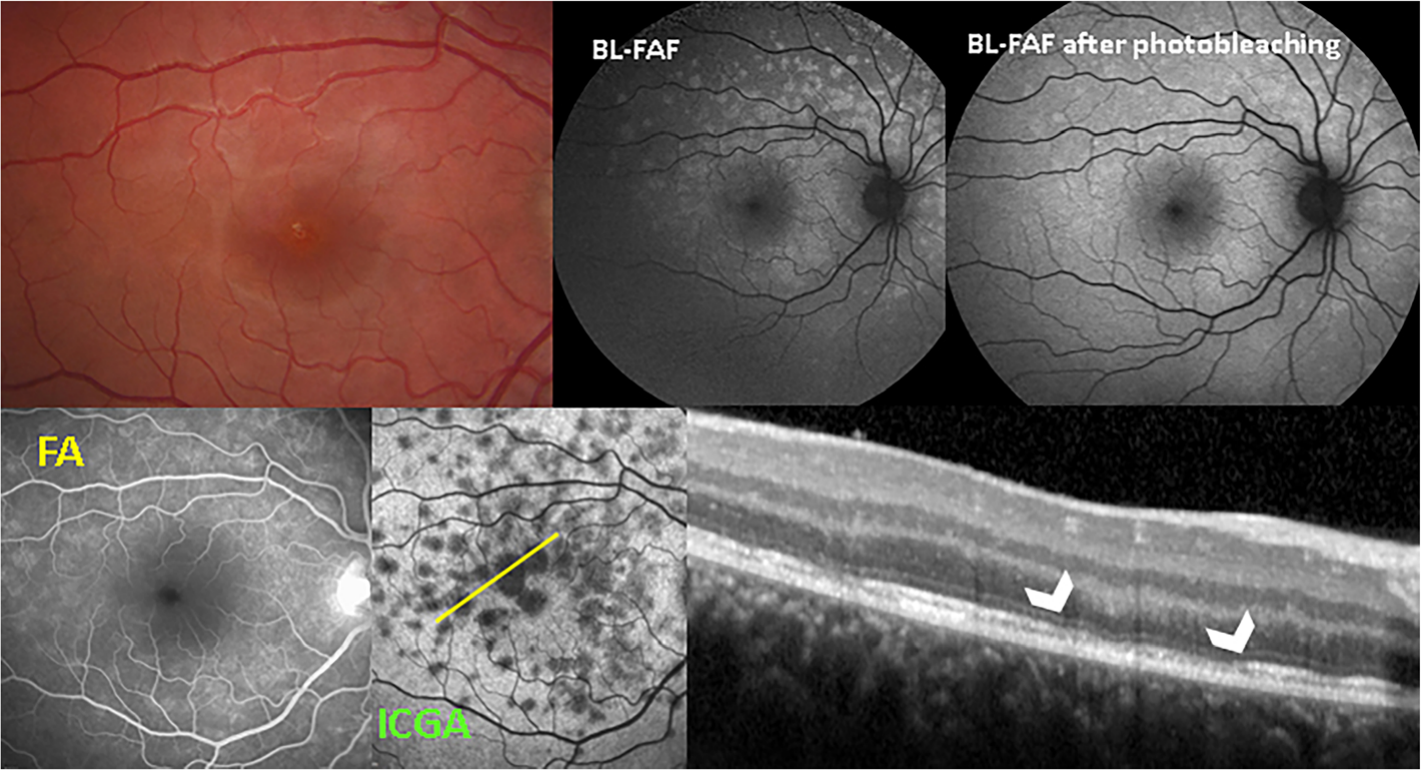
SD-OCT images in MEWDS. Fundoscopy and SD-OCT in a patient with foveal granularity. In the acute phase (top images) fundus photography reveals foveal granularity the only fundus finding. SD-OCT of the fovea shows attenuation of the ellipsoid zone (photoreceptors) (red arrow) and absence of the interdigitation zone (blue arrow). In the convalescent phase (bottom images) fundus photography and SD-OCT are back to normal

The SD-OCT appearance of MEWDS is that of disruption mainly of the ellipsoid zone (EZ-photoreceptor outer segments) and interdigitation zone (IZ) complex in the fovea (Fig. 10) and outside it is sometimes associated with reflective focal lesions that crossed the external limiting membrane line. As these lesions are very demonstrative and cause impressive secondary signs such as BL-FAF hyperautοfluorescence many reports situate the origin of the disease in these structures, although they only represent the consequence of ischaemia due to choriocapillaris non-perfusion [3, 7, 30]. The peripheral lesions consist of larger EZ discontinuity or disruption defined “spots”. The spots have been recognized with adaptive optics scanning laser ophthalmoscopy (AOSLO) as areas with absence of the photoreceptor outer segments [55, 56]. All the foveal and peripheral lesions spontaneously resolve with time with small areas of focal RPE atrophy in the most severe cases [57]. SD-OCT combined with decreased near-infrared fundus autofluorescence (NIR-FAF) was shown to characterise foveal granularity [2]. The choroidal thickness can increase in the acute phase of MEWDS and go back to normal in the recovery phase [58].

### Blue-light fundus autofluorescence (BL-FAF)

**Fig. 11:**
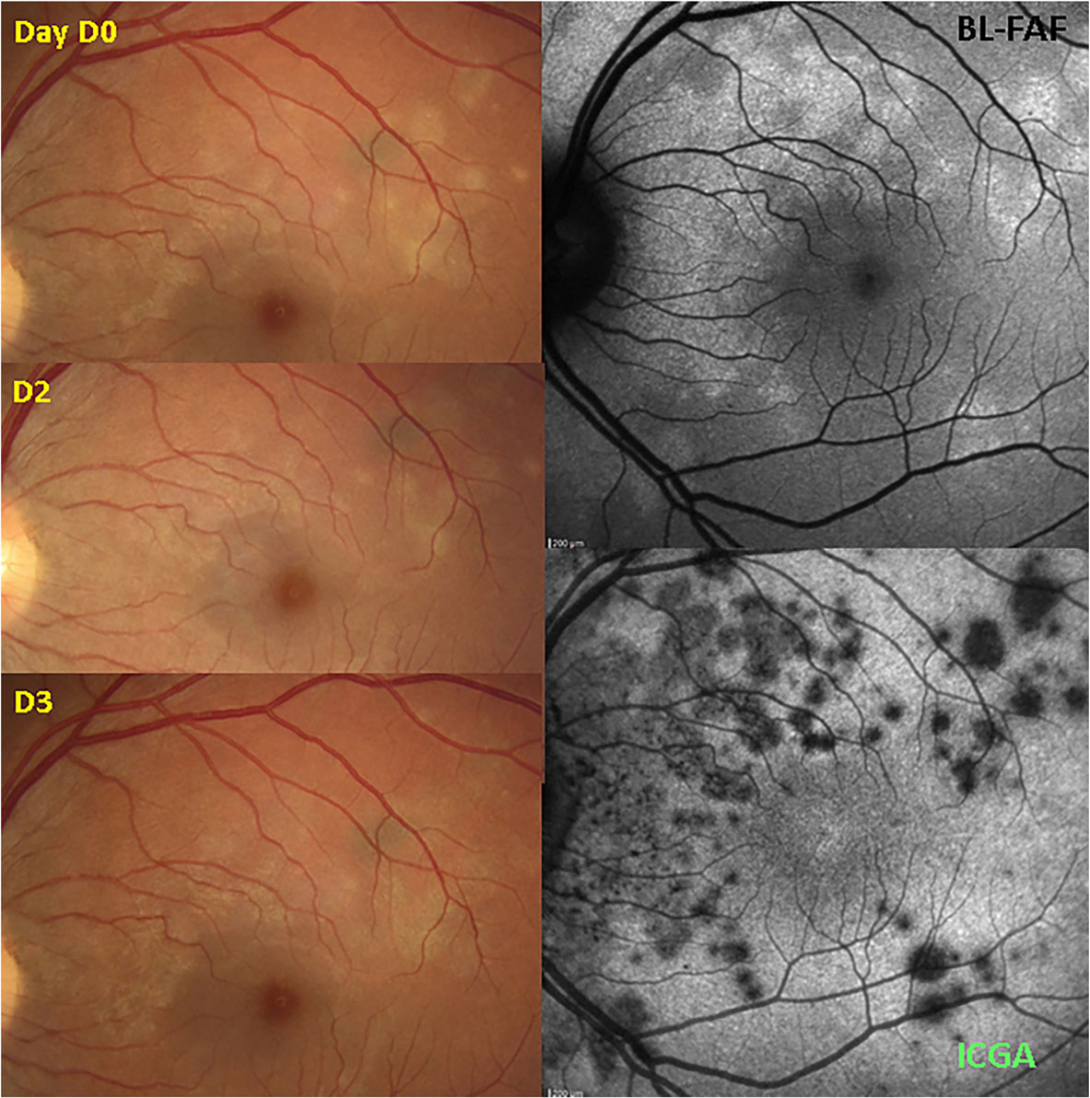
SD-OCT / BL-FAF / FA / ICGA signs of MEWDS. Patient with a 4-day history of photopsias in right eye. Fundus photography does not show any pathology. BL-FAF shows areas of hyperautofluorescence due to loss of photoreceptor outer segments. Photobleaching performs loss of photopigment in the rest of the fundus (top right). FA (bottom left) shows very faint hyperfluorescence while ICGA (bottom middle) clearly delineates diseased areas. SD-OCT shows interruption of the ellipsoid zone (arrowheads) corresponding to the ICGA hypofluorescent areas

BL-FAF imaging is a technique that became available recently for assessing the RPE function and the integrity of the chorioretinal interface. It is generated from the bisretinoids of lipofuscin in the RPE cells [59]. This material, the major fluorophores in the eye, is a mixture of several bisretinoids (A2E, A2PE) that are by-products of the visual cycle. These bisretinoids form primarily in the photoreceptor outer segments and are deposited secondarily in the RPE cell lysosomes during the process of photoreceptor outer segment phagocytosis [60, 61]. The intensity of the autofluorescent signal is modified by the variation of the amount of fluorophores and by the absorption of light by macular pigments and by photopigments in the photoreceptor outer segments [62]. Under normal conditions, visual photopigments absorb the exciting blue light, thereby attenuating the autofluorescent signal coming from the RPE. In MEWDS, active disease is reflected by bright autofluorescent patterns that become isoautofluorescent after photobleaching with blue light [63] (Fig. 11).

**Fig. 12:**
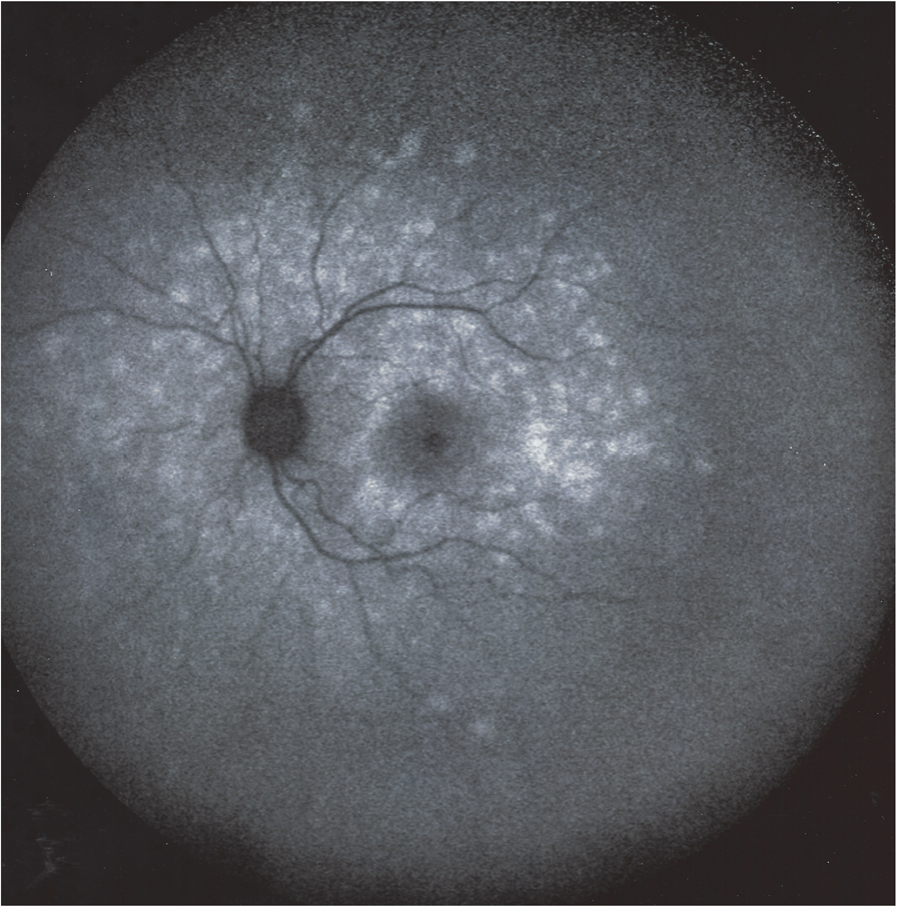
BL-FAF wide-field in a typical MEWDS case. Global display of hyperautofluorescent area on a wide-field picture

This indicates that the hyperautofluorescence originates from the loss of photoreceptor outer segments with consequent reduction of the photopigment density causing a better visualization of natural background autofluorescence [64]. This is especially significant because sometimes fundoscopy shows absence of pathology (Figs. 3 & 11) and fluorescein angiography may not be relevant. BL-FAF imaging represents a useful, fast, non-invasive and very sensitive diagnostic technique to evaluate inflammatory disorders affecting the chorioretinal interface. Wide-field BL-FAF is useful to give global view of the diseased areas (Fig. 12). The fact that BL-FAF exactly co-localises with ICGA hypofluorescent areas, indicating non-perfusion, supports the fact that photoreceptor outer segment damage results from consequent ischaemia.

### Optical coherence tomography angiography (OCT-A)

Since several years optical coherence tomography angiography has been proposed to examine several intraocular vascular pathologies including choriocapillaritis entities characterised by choroidal/choriocapillaris non-perfusion which appeared on OCT-A as vascular drop-outs. For MEWDS, several reports seemed to indicate that the choriocapillaris was intact and hence these reports concluded that the only damaged structure was the outer retina [30, 65, 66]. The principle on which OCT-A is based on is called diffractive particle movement detection performed by sequential OCT B-scans, so identifying vascular flow. However, in end-capillaries, suspected to be at the origin of MEWDS, there is practically no flow and capillary drop-out cannot be detected as it is possible for choriocapillaritis entities such as APMPPE where larger vessels are involved [67]. Even in MEWDS the degree of vascular choroidal non-perfusion can differ from one case to another and there are also reports of capillary drop-outs probably found in more severe cases of MEWDS [51, 68]. On the other hand, absence of perfusion and hence absence of the ICG dye in these areas can perfectly well be identified by ICGA. Therefore, unlike in other choriocapillaritis entities such as APMPPE, MFC and serpiginous choroiditis (SC), OCT-A is probably inappropriate for most MEWDS cases, as it is unable to detect whether there is choriocapillary drop out or not.

## Clinicopathology of MEWDS

MEWDS has been included in the past in a nebula called “white dot syndromes” (WDS), simply based on the resembling appearance of the fundus aspect of the diverse disease entities included by the first promoters of this terminology [21]. In those times it was a meritorious effort to attempt to bring more clarity in these clinical entities difficult to understand. However, this purely phenomenological approach brought together disease entities that, apart from a similar aspect, had nothing in common. This approach was understandable since the tools were not available for a more detailed analysis of the actual clinicopathology of these diverse diseases. The methodology applied, based on observation, had prevailed for years, and was at the origin of so many accurate disease characterisations, describing new individual disease entities [14, 17, 19, 22], but was inappropriate when using it to attempt to explain disease mechanisms and disease classifications. Diseases listed in the group of WDSs in 1995, all choroidal inflammatory diseases, were included at a time when imaging exploration of the choroid was still very limited. In the mid-1990s, ICGA became available and allowed to investigate more precisely the choroid and understand clinicopathological mechanisms of choroidal inflammatory diseases. ICGA allowed to sort out diseases predominantly involving the choroidal stroma, such as Vogt-Koyanagi-Harada (VKH) disease and HLA-A29 birdshot retinochoroiditis on one side and those that involved predominantly the choriocapillaris such as MEWDS, APMPPE, MFC and SC on the other side [24]. Several publications, past and present, classified MEWDS in the sub-group of primary inflammatory choriocapillaropathies [13, 69, 70]. In 2016, a divergent hypothesis on the pathogenetic mechanism of MEWDS was proposed, speaking of a primary “photoreceptoritis” [30]. Indeed, the photoreceptor layer is damaged in MEWDS and photoreceptor loss is at the origin of the typical BL-FAF hyperautofluorescent areas seen on BL-FAF, characterising MEWDS. However, this layer is only damaged secondarily, due to choriocapillaritis. The assumption of a primary photoreceptoritis was based on a misinterpretation of ICGA findings by the authors and on an alleged integrity of the choriocapillaris on OCT-A, which was rectified in an editorial [71]. In fact, OCT-A which is based on flow do not detect end-capillary low flow vessels of the choriocapillaris and hence is unable to detect whether there is flow or not, while ICGA is able to detect non-perfusion by showing ICG hypofluorescence [71]. Depending on the extent of choriocapillaris involvement, a recent report showed that there was indeed choriocapillaris drop-out when analysed by Swept Source OCT-A [68]. Another report identified choriocapillaris flow deficit in MEWDS patients and interestingly showed that in 7/34 patients with overlapping multifocal choroiditis were identified to have these changes, speaking for a common mechanism in these two choriocapillaritis entities [72]. Moreover, there is a variability of degrees of choriocapillaris involvement in MEWDS cases determining diverse OCT-A findings and different FA and ICGA features depending on the severity degree [73].

**Fig. 13:**
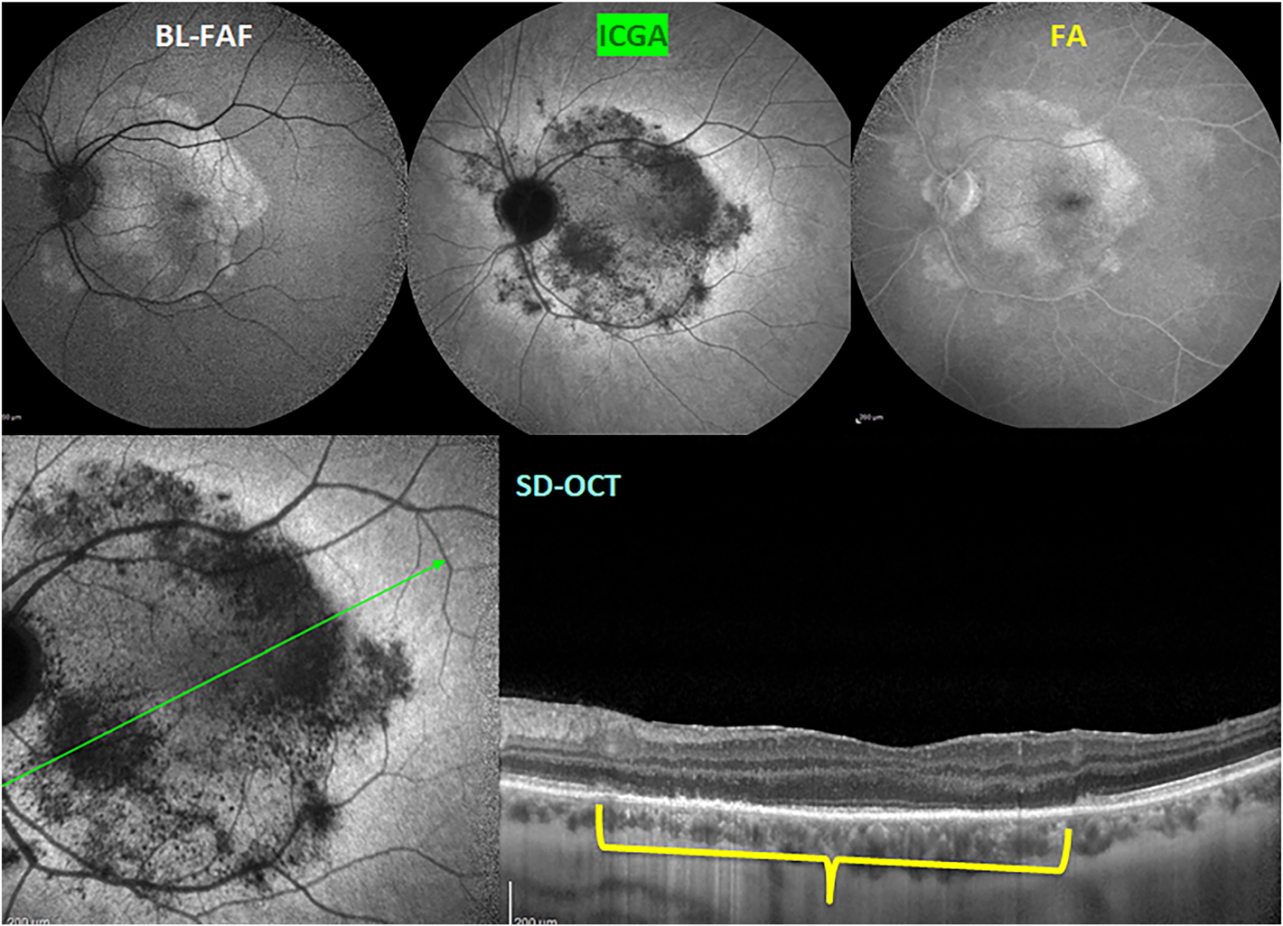
Masquerade of MEWDS. Case of acute syphilitic posterior placoid mimicking MEWDS. BL-FAF (top left) shows a placoid area of hyperautofluorescence. ICGA late frame (top middle) shows a hypofluorescent dark area due to choriocapillaris hypo and/or non-perfusion. FA late frame (top right) shows retinal staining probably caused by reactive exudation due to ischaemia of external retina. SD-OCT scan through macular area shows loss of photoreceptor outer segments (yellow bracket) Green line on ICGA frame (bottom left) shows the orientation of the SD-OCT scan (bottom right)

There are additional arguments to those exposed in the editorial by Lages et al., that are speaking for primary choriocapillaritis in MEWDS [71], including the implication of the choroid in MEWDS shown in numerous reports that cannot be ignored [58, 74–76]. Furthermore, diverse vaccinations were shown to trigger both MEWDS [77] and APMPPE cases [78]. It is most improbable that a vaccine induced immune reaction would directly target the photoreceptors in one case (MEWDS) and the choriocapillaris in the other (APMPPE). The most commonly suspected mechanism is an immune induced choriocapillaritis in both cases, which, following pioneering pragmatism, is also the mechanism suspected in MEWDS and other choriocapillaritis cases often preceded by flu-like viral symptoms. In a recent case report, interestingly, a patient initially diagnosed as MEWDS turned out to be an acute syphilitic posterior placoid chorioretinopathy (Fig. 13) (ASPPC) [79], a disease characterised by damage to photoreceptors due to choriocapillaritis [46].

After the publication of Pichi et al., several publications interpreted photoreceptor damage, a real and constant finding in MEWDS, as a primary involvement. All these studies failed to perform ICGA that could have shown choriocapillaris nonperfusion not apparent on OCT-A [33, 66]. In contrast, a perfect example of primary outer retinal, photoreceptor disease (photoreceptoritis) is represented by acute zonal occult outer retinopathy (AZOOR) [80, 81].

## Differential diagnosis and practical diagnostic criteria of MEWDS

MEWDS is a disease sometimes difficult to diagnose. As its name indicates, the characteristic fundal white dots are evanescent and may not be present any more when the patient consults with some delay after the onset of symptoms. Several clinical elements are helpful in the diagnosis, including a flu-like viral episode preceding symptoms (photopsias and subjective scotomas), fundal yellow-white dots, granularity of the fovea, very variable decrease of visual acuity and visual field impairment that can both be very severe depending on the degree of severity of choriocapillaris non perfusion [1, 2, 9]. Multimodal imaging has contributed significantly to an easier diagnosis. Fluorescein angiography, showing discreet hyperfluorescence in the involved areas is not the most useful method characterising MEWDS. However, the combination of ICGA, BL-FAF and SD-OCT represent an extremely forceful triad complementing clinical findings in the diagnosis of MEWDS [82]. ICGA hypofluorescence was found to delineate exactly the diseased areas indicating choriocapillaris hypo or non-perfusion. Interestingly, these hypofluorescent areas are still detectable when patients consult late, after the fundus lesions have vanished, allowing to make the diagnosis retroactively [6, 7, 83]. Since BL-FAF has come into use, BL-FAF-hyperautofluorescence marked the diseased areas co-localising with ICGA hypofluorescence and proved to be a marker at least as good as ICGA to detect MEWDS lesions, being especially useful for the follow-up as it is a non-invasive modality [84–86]. Hyperautofluorescence on BL-FAF is explained by the loss of the photoreceptor photopigment screen unmasking the normal underlying RPE autofluorescence [82]. This morphological change is apparent on SD-OCT, showing loss of photoreceptor outer segments corresponding to the areas where BL-FAF hyperautofluorescence and ICGA hypofluorescence are localised [87, 88]. Based on these imaging modalities clear diagnostic criteria can be elaborated (Table 2).

**Table 2:** Diagnostic criteria for MEWDS

1. Photopsias and/or subjective scotomas at or prior to presentation and/or preceding flu-like episode^a^	
2. Triad of ICGA hypofluorescent and BL-FAF hyperautofluorescent areas and corresponding loss or damage of ellipsoid zone on SD-OCT^a^	
3. Multiple yellow-white fundus dots and/or foveal granularity^b^ (helpful but moderate to low sensitivity)	
4. Unilateral clinical involvement^b^ (helpful)	
5. Exclusion of other infectious, inflammatory or masquerading entities	
6. Scotomas and/or enlarged blind spot-on visual field	
7. Often young myopic women^b^	

^a^Essential needed criteria

^b^Helpful criteria

Recently, “classification criteria” resulting from a sophisticated, “machine learning” and statistical process, have been proposed [31]. This is an appreciable intellectual effort but to no avail, similar to other “classification criteria” of other posterior uveitis entities reported by the same group because they lack practical usefulness as both ICGA and BL-FAF, the most sensitive modalities to detect MEWDS lesions, failed to be included. SD-OCT, as explained in this attempt, can indeed show the lesions at the level of the outer retina but the pattern of lesions needs to be globally displayed by ICGA, and/or by BL-FAF in case of unavailability of ICGA. In practice, these two determining imaging modalities are essential today to diagnose MEWDS and other choriocapillaritis entities [24] and they have been omitted in the recently proposed classification criteria based on a quite limited number of cases [31].

The main clinical differential diagnosis that the practitioners may encounter is retrobulbar optic neuritis in case of extensive visual field deficit. We received more than one referred patient with this diagnosis. A typical case history is exposed on Fig. 1. This patient was sent with the diagnosis of retrobulbar neuritis. When applying the diagnostic triad of ICGA, BAF and SD-OCT the diagnosis of MEWDS was easily performed (Fig. 1). Another differential diagnosis within choriocapillaritis entities is idiopathic multifocal choroiditis (MFC), as MEWDS and MFC have the same features using the diagnostic triad of ICGA, BAF and SD-OCT [88, 89]. MFC can present initially as MEWDS and when the disease evolves with recurrences or becomes bilateral with chorioretinal scars the diagnosis has to be redirected towards MFC [12]. It is difficult to make the difference between whether MEWDS and MFC are overlapping syndromes or whether the first MFC episode presents with MEWDS features, only the evolution permitting to specify the ultimate diagnosis [12]. Acute idiopathic blind spot enlargement (AIBSE) has been linked to MEWDS. The visual field changes have been found to correspond to peripapillary hypofluorescence as those seen in MEWDS when ICGA was performed [25, 27, 90, 91]. The topic on overlapping and masquerade syndromes will be dealt with in more details in the next section.

## Overlapping syndromes and masquerades of MEWDS

An overlap between MEWDS and other inflammatory choriocapillaropathies, especially MFC which includes punctate inner choroidopathy (PIC), has been reported involving the ipsilateral or contralateral eyes [12, 89]. Kang et al. [72] have recently compared the clinical characteristics of 27 patients with classic MEWDS and 7 patients with atypical MEWDS overlapping with MFC, involving the same eye in 5 and contralateral eye in 2 of them. While there was no significant difference in the demographic features, presenting symptoms, refractive error, intraocular inflammation, lesion distribution, or time to resolution, RPE hyperpigmentation and focal choroidal excavation were seen exclusively in those with overlapping MFC. Additionally, atypical cases had a thicker choroid at presentation, and the presence of subfoveal MFC significantly influenced the final visual acuity [72]. Essilfie et al. [92] have recently described 17 cases with “secondary” MEWDS, 15 of them having pre-existing or late onset MFC lesions. Final visual acuity was 20/20 in all cases, except 2 patients with macular CNVs [92].

Many conditions that masquerade as MEWDS have been reported principally based on clinical examination [93]. However, when considering strict diagnostic criteria including, in addition to clinical signs and symptoms, unilaterality, the triad of ICGA, BL-FAF and SD-OCT, in several of these conditions MEWDS can be ruled. For instance, AZOOR which shows similar BL-FAF and SD-OCT signs has preserved choriocapillaris not presenting ICGA hypofluorescent areas [81]. Of the 13 patients reported as masquerading MEWDS, two conditions, syphilis, in particular ASPPC, and MFC, occurring in five reported patients, cause a real differential diagnostic problem as the clinicopathological mechanism is comparable [46, 72, 79].

## Conclusion

Today MEWDS can be diagnosed with a high degree of certainty thanks to more recent imaging modalities such as ICGA, BL-FAF and SD-OCT in complement of the clinical examination. The diagnostic criteria as exposed in this article represent a highly practical tool not only to diagnose MEWDS but also to follow the evolution of lesions. The latter point is important, as, albeit MEWDS has usually a spontaneously favourable evolution, the diagnostic and monitoring approach exposed here can spot the rare cases which do not have a resolutive evolution or which have an overlapping evolution to MFC. Such precise diagnostic criteria are also practically helpful to exclude other diagnoses such as retrobulbar neuritis which is often posed in non-diagnosed MEWDS cases.

## Abbreviations

MEWDS: Multiple evanescent white dot syndrome
BL-FAF: Blue-light fundus autofluorescence
ICGA: Indocyanine angiography
SD-OCT: Spectral domain optical coherence tomography
IS/OS: Inner segment/ outer segment
OCT-A: OCT angiography
MFC: Idiopathic multifocal choroiditis
APMPPE: Acute posterior multifocal placoid pigment epitheliopathy
ASPPC: Acute syphilitic posterior placoid chorioretinitis
AZOOR: Acute zonal occult outer retinopathy
RPE: Retinal pigment epithelium
AMIC: Acute multifocal choriocapillaritis
WDS: White dot syndromes
AIBSE: Acute idiopathic blind spot enlargement
CNVs: Choroidal neovascularisation
VF: Visual Fields
IZ: Interdigitation zone
EZ: Ellipsoid zone
AOSLO: adaptive optics scanning laser ophthalmoscopy
NIR-FAF: Near-infrared fundus autofluorescence
SC: Serpiginous choroiditis
PICCPs: Primary inflammatory choriocapillaropathies
VKH: Vogt-Koyanagi-Harada
FA: Fluorescein angiography
PIC: Punctate inner choroidopathy
